# Novel Molecular Challenges in Targeting Anaplastic Lymphoma Kinase in ALK-Expressing Human Cancers

**DOI:** 10.3390/cancers9110148

**Published:** 2017-10-28

**Authors:** Abdulraheem Alshareef

**Affiliations:** 1 Department of Clinical Laboratory Sciences, College of Applied Medical Sciences, Taibah University, Almedinah, Medina P.O. Box 41477, Saudi Arabia; amshareef@taibahu.edu.sa or al15@ualberta.ca; 2 Department of Laboratory Medicin and Pathology, University of Alberta, Edmonton, AB T6G 2E1, Canada

**Keywords:** anaplastic lymphoma kinase, ALK-expressing cancers, cancer stem cells, crizotinib, clinical resistance, tyrosine kinases, tyrosine kinase inhibitors

## Abstract

Targeting anaplastic lymphoma kinase (ALK), a receptor tyrosine kinase receptor initially identified as a potent oncogenic driver in anaplastic large-cell lymphoma (ALCL) in the form of nucleophosmin (NPM)-ALK fusion protein, using tyrosine kinase inhibitors has shown to be a promising therapeutic approach for ALK-expressing tumors. However, clinical resistance to ALK inhibitors invariably occurs, and the molecular mechanisms are incompletely understood. Recent studies have clearly shown that clinical resistance to ALK inhibitors is a multifactorial and complex mechanism. While few of the mechanisms of clinical resistance to ALK inhibitors such as gene mutation are well known, there are others that are not well covered. In this review, the molecular mechanisms of cancer stem cells in mediating resistance to ALK inhibitors as well as the current understanding of the molecular challenges in targeting ALK in ALK-expressing human cancers will be discussed.

## 1. Introduction

**Figure 1:**
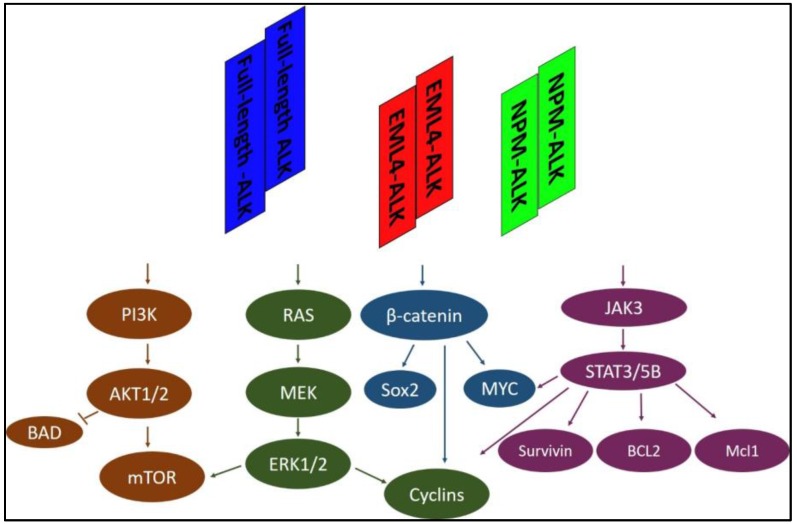
Representative signaling pathways activated by full-length ALK, EML4-ALK, or NPM-ALK. The ALK protein interacts and activates many essential adaptors involved in multiple signaling pathways, including PI3K, RAS/MEK/ERK, β-catenin, and JAK/STATs. Only four representative signaling pathways are shown here. EML4-ALK: echinoderm microtubule-associated protein like 4-anaplastic lymphoma kinase; NPM-ALK: Nucleophosmin-anaplastic lymphoma kinase; STAT: Signal transducer and activator of transcription; PI3K: phosphatidylinositol 3 kinase; ERK: extracellular signal-related kinase; JAK3: Janus kinase 3; Bcl2: B-cell lymphoma 2; Mcl1: Myeloid cell lymphoma 1; BAD: Bcl-2-associated death promoter; mTOR: mammalian target of rapamycin; MEK: MAPK (Mitogen-activated protein kinase)/ERK (extracellular signal-regulated kinase); Sox2: (sex determining region Y)-box 2.

Tyrosine kinases, such as ALK, are a very attractive therapeutic target for cancer treatment, especially on the basis of promising results from preclinical and early clinical studies [1]. Anaplastic lymphoma kinase (ALK) is a receptor tyrosine kinase that was initially discovered and characterized in a rare type of lymphoma called anaplastic large-cell lymphoma (ALCL) as an NPM-ALK fusion protein [2]. Specifically, the catalytic domain of the ALK protein was fused with the amino terminus of nucleophosmin (NPM), and it was found that the NPM-ALK fusion protein resulted in the constitutive activation of the ALK tyrosine kinase, thereby leading to deregulation of multiple cell signaling pathways and increased tumorigenicity (Figure 1) [3,4,5]. Subsequent studies of ALCL and other types of human cancer have revealed additional fusion partners of ALK and various types of *ALK* gene aberrations [6,7]. For example, the echinoderm microtubule-associated protein like 4 (*EML4*)-*ALK* fusion was identified in ~5% of non-small cell lung cancers (NSCLC) [8,9]. Amplified *ALK* or mutated *ALK* was identified in ~14% of neuroblastomas (NB), the most common and aggressive childhood malignancy [10,11,12,13]. To date, several ALK inhibitors are at various stages of clinical testing and the US Food and Drug Administration (FDA) [1]. Although most clinical results regarding ALK inhibitors are from patients with ALK-positive non–small-cell lung carcinoma (NSCLC), it is clear from preclinical studies that ALK inhibition is effective in all ALK-expressing cancers [14]. 

The data collected from clinical studies, especially for crizotinib (the first ALK inhibitor used in the clinic), were extremely promising [1]. In ALK^+^ NSCLC, for instance, comparing crizotinib with standard chemotherapy in the second-line setting resulted in an improved overall response rate (65% vs. 20%, respectively), a shorter response time (6.3 vs. 12.6 weeks), and an improved median progression-free survival (7.7 vs. 3.0 months) with crizotinib [15]. In ALK^+^ ALCL patients, crizotinib was administered to seven adults with resistant high-stage disease and resulted in a complete response (CR) in three patients and a partial response in one patient [16]. This later study was expanded and had a total of 11 patients (9 with ALCL) and a CR was observed in all 9 patients [17]. Furthermore, the Children’s Oncology Group-sponsored Phase 1 clinical trial (NCT00939770) with crizotinib in children with refractory ALK^+^ ALCL resulted in a CR in eight of the nine patients [18]. This Phase 1 clinical trial included 34 NB patients with recurrent or refractory cancer, and showed a wide range sensitivity to ALK kinase inhibition [18]. Specifically, only 2 out of 34 (6%) patients showed complete remission, 8 (23.5%) showed stable disease while 24 (71%) showed progressive disease [18].

**Figure 2:**
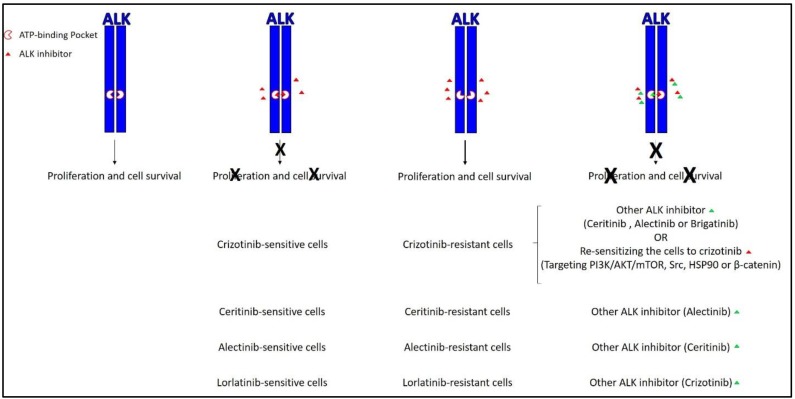
The current active approaches to overcome resistance to ALK inhibitors. The most common approach mainly relies on second and third generation ALK inhibitors such as ceritinib, alectinib, and brigatinib. The less common approach relies on re-sensitizing resistant cells to ALK inhibitors by targeting other signaling pathways. X represents the inhibitory effect of the ALK inhibitor. Green triangle represents the addition of another ALK inhibitor. PI3K: Phosphoinositide 3-kinase; HSP90: heat shock protein 90.

Resistance to ALK inhibitors, including even second- or third-generation drugs used as a single therapy, is a ubiquitous problem in ALK-expressing cell lines as well as treated patients (Table 1 and Figure 2) [1]. Resistance to crizotinib, for instance, was initially reported in NSCLC [15,19] and inflammatory myofibroblastic tumors [20], followed by NB [18] and ALCL [17]. Previous reports have generally suggested two categories of mechanisms of resistance: (1) resistance mediated by mutations in the ALK kinase domain impairing binding of an inhibitor to an ALK protein, and/or (2) the activation of compensatory alternative oncogenic drivers such as MET, epidermal growth factor receptor (EGFR), KRAS, and c-KIT [1]. However, there is a lack of knowledge on the molecular basis of this resistance. In other words, almost all of the previous studies have focused on acquired resistance (which is caused by post-treatment changes such as alteration in drug targets and the activation of compensatory survival signaling pathways), while knowledge on intrinsic resistance (which includes the factors that exist before treatment such as the presence of cancer stem cells) is almost lacking in ALK^+^ cancers. These two mechanisms of resistance have been previously reviewed in [21,22]. In this review, the role of cancer stem cells and how it impacts on the resistance to ALK inhibitors as well as the current understanding of the molecular challenges in targeting ALK in ALK-expressing human cancers will be discussed.

## 2. Reported Mechanisms of Resistance

Resistance to targeted therapy has been reported to be mediated through multiple mechanisms [1]. Some of these mechanisms are known to exist among almost all tyrosine kinases, including ALK. For example, gene amplification, gene mutation, and upregulation of alternative signaling pathways; all of which have been shown to induce drug resistance [22,23,40]. Unlike ALK, there are several mechanisms of resistance that have been reported in EGFR-expressing lung cancer cells and BCR-ABL (breakpoint cluster region-abelson)-expressing chronic myeloid leukemia (CML) cells. The existence of cancer stem cells (CSCs) is one of these mechanisms. For instance, stem cell population in lung cancer cells were shown to be relatively resistant to gefitinib, a tyrosine kinase inhibitor showing specificity to the epidermal growth factor receptor (EGFR) [41]. One study showed that acquired resistance to gefitinib was associated with a manifestation of stem cell–like properties in cancer cells [42]. Similarly, resistance to imatinib, a tyrosine kinase inhibitor showing specificity to BCR-ABL, has been well documented in leukemic stem cells. 

Mechanistically, leukemic stem cells have been shown to be attributed to imatinib resistance through three independent mechanisms. First, leukemic stem cells were found to express a relatively high level of BCR-ABL [43,44]. Second, leukemic stem cells were found to be inefficient in maintaining the intracellular accumulation of imatinib, mainly due to the relatively low expression of the organic cation transporter-1 (OCT1), which is responsible for the cellular uptake of imatinib [45], as well as the relatively high expression of ABCB1 (ATP Binding Cassette Subfamily B Member 1) that mediates the efflux of imatinib [46]. Third, imatinib resistance was shown to be mediated by the activation of alternative signaling pathways such as Mitogen-activated protein kinase (MAPK), Notch, and hedgehog that maintain viability and growth despite continued suppression of BCR-ABL kinase activity [47,48,49,50].

The other mechanism of resistance that has been previously reported is histologic transformation which includes epithelial mesenchymal transition (EMT) and small cell transformation [40,51]. For instance, many studies have reported that histologic transformation, mainly EMT, could induce cancer cell resistance to EGFR-tyrosine kinase inhibitors (TKIs) [52,53,54,55]. Specifically, the transformation to SCLC as acquired resistance mechanism is observed in 14% of TKI-treated EGFR mutant pulmonary adenocarcinomas [56,57,58]. In ALK^F1174L^-driven NB cells, ALK inhibitor resistance was found to be associated with the induction of EMT [59]. Importantly, co-targeting EGFR (in lung cancer) or ALK (in NB) and the molecules that drive these pathways can reverse TKIs resistance [59,60,61,62]. A more recent study has suggested that EMT is only associated with, but does not drive resistance to, ALK inhibitors among EML4-ALK^+^ NSCLC [63].

Furthermore, it has been reported that due to a heterogeneous population of cancer cells, some of the population are not fully dependent on the activity of tyrosine kinases for their survival, which may be the reason behind the insensitivity of these cells to TKIs [64]. For instance, leukemia stem cells have been shown to utilize signaling pathways independent of BCR-ABL kinase activity for their maintenance and survival, and that these cells were insensitive to TKIs such as imatinib or dasatinib [65,66].

## 3. Role of Intra-Tumoral Heterogeneity in Dictating Resistance to ALK Inhibitors in ALK-Expressing Cancers

As mentioned previously, CSCs were shown to be a major contributing factor to disease relapses upon TKIs treatment. Oh et al., (2015) showed that targeting stemness with rapamycin, an mTOR inhibitor, synergized the crizotinib effect in EML4-ALK^+^ cells (lung cancer) in vitro and in vivo [67]. Additionally, they showed that the rapamycin treatment sensitized the crizotinib-resistant cell line to ALK inhibition [67]. A more recent study showed the synergistic effect of ALK and mTOR inhibitors in the treatment of NPM-ALK^+^ cells (ALCL) [68]. Using an mTOR inhibitor, namely Torin2, which is a selective mTORC1 inhibitor, was also shown to restore sensitivity to crizotinib in ALK-mutated (ALK^F1174L^) NB cells [28,69]. These studies have shed light into the importance of CSCs in ALK-expressing cancer cells; however, they did not mechanistically study CSCs as none of these studies purified and studied CSCs. 

In our recent study, we found that CSCs derived from NB cells were significantly more resistant to crizotinib (under review). Of note, NB CSCs were purified based on their responsiveness to a Sox2 reporter, a strategy that has been used previously for several different cancer models [70,71,72,73,74,75]. Importantly, we concluded that the crizotinib resistant phenotype in CSCs can be attributed to their high β-catenin expression since siRNA knockdown of β-catenin sensitizes CSCs to the crizotinib treatment. Our data suggested that combining β-catenin inhibitors and ALK inhibitors may be useful in treating NB patients. To the best of our knowledge, this latter study is the only study, to date, that has investigated the role of CSCs as a contributing factor to treatment failure and disease relapses to ALK inhibitors. Further studies need to be performed to study the CSCs and their role in mediating ALK TKIs resistance.

## 4. Role of ALK-Interacting Proteins in Mediating TKIs Resistance

Tyrosine kinases are known to bind to a large number of cellular proteins, thereby mediating their oncogenic effect [4]. To date, there is little known about whether some of these binding proteins might play a role in modulating resistance to TKIs. For instance, targeting β-catenin has been shown to cause an abrogation of tyrosine kinase resistance in the BCR-ABL CML model [76]. In a lung cancer model, inhibition of β-catenin was shown to enhance the anticancer effect of EGFR-TKI in EGFR-mutated cells [77]. Of note, β-catenin is the central mediator of Wnt/β-catenin signaling, and it can be localized either in the adherens junctions and is involved in cell–cell contacts, or in the nucleus where it is implicated in transcriptional regulation and chromatin modification [78]. The mechanism of how β-catenin mediates resistance to these TKIs.

Work from our laboratory using the NPM-ALK^+^ ALCL and ALK^+^ NB cells showed a physical interaction between ALK and β-catenin [32,79]. Computational analysis could provide a starting point for more in-depth mechanistic understanding and explanation for the reason behind ALK TKIs resistance and predicting the proteins that could mediate the blockage of TKI-ALK binding. To date, the fate of ALK—β-catenin interaction and other ALK-interacting proteins is still minimally understood, and requires more mechanistic studies.

## 5. CETSA as a Tool That Can Be Used to Predict the Resistance to ALK Inhibitors

The current read-out used to measure the effect of ALK inhibitors focuses on phenotypic assays where the response to an inhibitor is based on a functional readout such as changes in the phosphorylation status of downstream targets, or the impact on cellular viability [80]. While these functional readouts are very useful, they do not provide sufficient information regarding the resistance. We have recently shown that CETSA (Cellular Thermal Shift Assay), a recently described method that allows for the rapid and simple assessment of drug target engagement in a cellular context [81,82,83], is useful in predicting crizotinib sensitivity in ALK-carrying cancer cells [32]. Previous studies have shown that CETSA is as an excellent tool to evaluate the physical binding of an inhibitor to its target in intact cells [81,82,83]. A few studies have used the CETSA assay to evaluate crizotinib treatment [32,84,85]. The first report assessed the photosensitivity side effect of many kinase inhibitors, including crizotinib, on K562 (a BCR-ABL^+^ CML cell line) by combining the CETSA method with multiplexed quantitative mass spectrometry (MS) [84]. In the second report, the authors demonstrated a bond between the (S)-enantiomer crizotinib (which is not the clinically used ALK inhibitor; (R)-enantiomer) and MTH1 (MutT Homolog 1) and thereby worked as a suppressor of MTH1 activity [85]. In the third study, the authors identified a significant positive correlation between crizotinib-ALK binding and the observed IC_50_, which provides a logical justification for the differential responsiveness [32]. Additionally, it proved that the CETSA assay was a very useful tool to predict crizotinib sensitivity in different ALK-carrying cancer types.

CETSA has increased in popularity as a tool to validate drug–target interaction [86,87]. For example, the proposed PARP-1 (Poly [ADP-ribose] polymerase-1) inhibitor iniparib reached Phase III clinical trials where it showed no efficacy, and was subsequently shown to lack activity against PARP-1 in living cells [86,87]. CETSA was used to compare the target engagement of PARP-1 for iniparib and olaparib [81], which is a well-established PARP-1 inhibitor in clinical development. Recently, the CETSA assay was used to assess the binding of these two PARP-1 inhibitors and showed that iniparib failed to induce a thermal shift, whereas olaparib binding induced a large thermal shift of PARP-1 [81]. Apparently, the mechanism of action of iniparib is not via physical binding to PARP-1; instead, iniparib may kill cancer cells by unspecific modification of cysteine residues. All these reports highlight the significance of implementing CETSA as a tool that can prove the TKI binding to its target and thereby predict resistance in early stages of treatment.

## 6. Potential Significance of Precursor mRNA in Mediating Resistance to ALK TKIs

Cancer cells are generally known to develop multiple mechanisms to escape the signaling inhibition caused by TKIs treatment. While the main mechanisms have been discussed earlier in this review, we recently postulated an additional mechanism where an ALK-intron retained transcript was detected in NB cell lines as well as patient samples (under review). This observation poses some intriguing questions with regard to the properties of ALK as an oncogene. In our hypothetical model, the *ALK-intron 19* (*ALK-I19*) transcript was the final precursor to the *fully spliced(FS)-ALK* transcript, whose high expression is fundamental for sustaining NB growth and proliferation. Perhaps due to this inherent dependency on ALK, many NB cells may pre-synthesize the *ALK-I19* transcript within the nucleus as a short-term storage system to bolster *FS-ALK* expression when it is critically required. This mechanism may be especially useful to the cancer cells to maintain homeostasis. In conditions of cellular stress, such as in normal tumor physiology like hypoxia and upon growth inhibiting drug treatments (i.e., chemotherapy), *ALK-I19* may increase *FS-ALK* levels. A comparable stress-induced mechanism was identified for the *ApoE* gene in central nervous system neurons [88]. Upon injury of the neuronal cells, an intron retained transcript of *ApoE* pre-synthesized in the nucleus enhanced the cytoplasmic level of fully spliced *ApoE* [88]. This mechanism leads to the immediate production of proteins upon cellular stress. This phenomenon of a ‘buffer’ transcript could be relevant given the vital importance of ALK activity in many cancer types.

What molecular factors are responsible for this phenomenon? Answering this important question could aid the development of more effective therapeutics for ALK^+^ patients. For example, Intron 4-retaining *CCDN1 (cyclinD1)* expressed in prostate and esophageal cancers was found to translate into a truncated cyclin D1 protein, which has oncogenic effects [89,90]. Indeed, RNA binding proteins, many of which participate in specific splicing complexes, are deregulated in cancers [91]. This disruption can lead to aberrant alternative splicing and boost tumorigenesis. Therefore, the examination of regulatory protein-networks involved in ALK protein synthesis and its role in mediating resistance to ALK TKIs is warranted.

## 7. Conclusions

This review presented data describing mechanisms of resistance to ALK TKI treatment and clearly showed that it is a multifactorial and complex mechanism (Table 1). There are at least four reasons supporting the notion that the mechanisms of resistance to ALK inhibitors are not exclusively attributed to one or two factors (e.g., specific ALK mutation). First, studies performed on crizotinib upon its discovery showed that a high concentration of crizotinib (defined as >300 nM) displayed off-target effects [92]; and the IC_50_ for U937, a histiocytic lymphoma cell line used as a negative control (as it expresses neither ALK nor c-Met) was 257 nM [93]. Second, NB cell lines carrying resistant ALK mutations such as ALK^F1174L^, displayed drastically different IC_50_ to crizotinib (i.e., IC_50_, 400 to 2000 nM)) [28]. Third, while some reports documented that crizotinib differential sensitivity in EML4-ALK-expressing cells was dependent on the EML4-ALK variant [94,95,96], other studies have shown no link between EML4-ALK fusion variants and crizotinib responses [97,98]. Fourth, crizotinib was shown to suppress the growth of ALK^+^ thyroid cancer cells; however, this potential therapeutic benefit was produced from non-Met/ALK-targeting effects [99]. All these facts highlight the importance of undertaking more research to understand the molecular basis of resistance to ALK TKIs.

The current active approach to overcome ALK-TKIs resistance mainly relies on second- and third-generation TKIs, with over 11 inhibitors being developed [100,101]. With increased experience in TKI resistance, the clinical response to the next-generation TKIs is commonly highly variable and unpredictable [101]. For instance, third-generation EGFR TKIs are being developed as part of a strategy to overcome treatment resistance to first- and second-generation EGFR TKIs in lung cancer patients [102]; however, resistance to third-generation EGFT TKIs such as AZD9291 and HM61713 are also being arise [103]. A pervading theme regarding resistance to TKI therapy is its mediation by secondary mutations, which has not been resolved by introducing new generations of more TKIs. For example, despite the clinical efficacy of the first-, second-, and third-generation BCR-ABL inhibitors, resistance occurs invariably and more than 50 distinct point mutations encoding single amino-acid substitutions in the kinase domain of the BCR-ABL1 gene have been detected in patients with imatinib-resistant CML [64,104]. Adding more inhibitors does not work, as previously shown for other tyrosine kinase inhibitors. Therefore, it is crucial to perform further research to understand TKI resistance in ALK^+^ cancer patients.

Taken together, the evidence presented in this review depicts the importance of continuous investigation towards a deeper understanding of clinical resistance. Although there are copious and complex questions to be solved, the recent advances in both clinical and preclinical research, facilitated by the impressive developments in experimental methods and techniques, has generated much enthusiasm and hope for the future.

## Figures and Tables

**Table 1:** Summary of first and next generation ALK inhibitors.

ALK Inhibitor	Other Names	FDA Approval (Month/Year)	Resistance Occurred [Reference]	Ways to Overcome Resistance [Reference]	
				Other ALK Inhibitors	Re-Sensitizing the Inhibitor
Crizotinib	PF-2341066 Xalkori^®^	Yes (08/2011)	Yes [15,23]	1. Ceritinib [24,25] 2. Alectinib [26] 3. Brigatinib [27]	1. Targeting PI3K/AKT/mTOR pathway [28,29] 2. Targeting Src [30] 3. Targeting HSP90 [31] 4. Targeting β-catenin [32]
Ceritinib	LDK-378 Zycadia^®^	Yes (04/2014)	Yes [33,34]	Alectinib [33,34]	Not performed
Alectinib	CH5424802 RO5424802 Alecensa^®^	Yes (12/2015)	Yes [35,36]	Ceritinib [35,37]	Not performed
Brigatinib	AP26113 Alunbrig™	Yes (04/2017)	Yes [38]	-	Not performed
Lorlatinib	PF-06463922	No	Yes [39]	Crizotinib [39]	Not performed

ALK: Anaplastic lymphoma kinase; FDA: Food and Drug Administration; PI3K: Phosphoinositide 3-kinase; mTOR: mammalian target of rapamycin; HSP90: heat shock protein 90.
